# Case Report: Tetralogy of Fallot in a Chinese Family Caused by a Novel Missense Variant of *MYOM2*

**DOI:** 10.3389/fcvm.2022.863650

**Published:** 2022-07-07

**Authors:** Jing Wang, Chunyan Wang, Haiyang Xie, Xiaoyuan Feng, Lei Wei, Binbin Wang, Tengyan Li, Mingan Pi, Li Gong

**Affiliations:** ^1^Department of Medical Genetics and Developmental Biology, School of Basic Medical Sciences, Capital Medical University, Beijing, China; ^2^Graduate School of Peking Union Medical College and Chinese Academy of Medical Sciences, Beijing, China; ^3^Center for Genetics, National Research Institute for Family Planning, Beijing, China; ^4^Department of Cardiothoracic Surgery, Wuhan Children's Hospital (Wuhan Maternal and Child Healthcare Hospital), Tongji Medical College, Huazhong University of Science and Technology, Wuhan, China; ^5^Department of Echocardiography, Wuhan Children's Hospital (Wuhan Maternal and Child Healthcare Hospital), Tongji Medical College, Huazhong University of Science and Technology, Wuhan, China

**Keywords:** Tetralogy of Fallot, whole exome sequencing, heterozygous variant, *MYOM2*, incomplete penetrance

## Abstract

**Background:**

Rare genetic variants have been identified to be important contributors to the risk of Tetralogy of Fallot (TOF), the most common cyanotic congenital heart disease (CHD). But relatively limited familial studies with small numbers of TOF cases have been reported to date. In this study, we aimed to identify novel pathogenic genes and variants that caused TOF in a Chinese family using whole exome sequencing (WES).

**Methods:**

A Chinese family whose twins were affected by TOF were recruited for this study. A WES was performed for the affected twins, their healthy brother, and parents to identify the potential pathogenic mutated gene(s). Heterozygous variants carried by the twins, but not the unaffected brother, were retained. Public databases were used to assess the frequencies of the selected variants, and online prediction tools were accessed to predict the influences of these variants on protein function. The final candidate variant was further confirmed by Sanger sequencing in other members of the family.

**Results:**

After several filtering processes, a heterozygous missense variant in the *MYOM2* gene (NM_003970.4:c.3097C>T:p.R1033C) was identified and confirmed by Sanger sequencing in the affected twins and their unaffected father, suggesting an inheritance pattern with incomplete penetrance. The variant was found to be extremely rare in the public databases. Furthermore, the mutated site was highly conserved among mammals, and as shown using multiple online prediction tools, this variant was predicted to be a detrimental variant.

**Conclusion:**

We assessed a family with TOF caused by a rare heterozygous missense variant of *MYOM2*. Our findings not only further confirm the significant role of genetics in the incidence of TOF but also expand the spectrum of the gene variants that lead to TOF.

## Introduction

Congenital heart diseases (CHDs) are the most common birth defects affecting approximately 1% of live births each year (1). Previous studies have shown that CHDs involve a variety of cardiac malformations including patent ductus arteriosus (PDA) and Tetralogy of Fallot (TOF) (2). The TOF, as a malformation of the cardiac outflow tract, comprises four major cardiac defects that occur together: ventricular septal defect, right ventricular hypertrophy, pulmonary stenosis, and aortic override (3). The incidence of TOF is about 0.04% worldwide (4). Researchers have suggested that the etiology of approximately 80% of patients with non-syndromic TOF is unexplained.

In recent years, increasing evidence indicates that TOF has high genetic heterogeneity and various genes involved in heart development may be responsible for the phenotype of the disease (5–7). Several previous studies have identified rare potential pathogenic variants of new candidate genes in patients with TOF using whole exome sequencing (WES) such as notch receptor 1 (*NOTCH1*) (7), GATA-binding protein 4 (*GATA4*) (8), NK2 homeobox 5 (*NKX2.5*) (9), Jagged 1 (*JAG1*) (10), forkhead box C2 (*FOXC2*) (11), T-box 5 (*TBX5*) (12), T-box 1 (*TBX1*) (13), and Fms-related tyrosine kinase 4 (*FLT4*) (7). These findings provide insights into the complex genetic variants responsible for TOF; however, the specific etiology of most TOF cases remains unknown. Therefore, further studies on the genetic etiology of TOF were needed to better understand its pathogenesis. In addition, most studies focused on sporadic cases of TOF, and reports on families with TOF patients were relatively limited (14–19).

In this study, we performed a WES analysis on a Chinese family with two TOF affected children to identify novel genetic causes of TOF. Through focused analysis of shared, likely deleterious variants, we found a heterozygous missense variant of Myomesin2 (*MYOM2*) (NM_003970.4:c.3097C>T:p.R1033C) in the affected twins. Two previous reports have described rare *MYOM2* variants in patients with TOF (20, 21). This study independently validates *MYOM2* variants in the Chinese population and provides further evidence of the importance of this gene in the incidence of TOF.

## Materials and Methods

### Subjects

A Chinese family with two affected fraternal twins was recruited from the Wuhan Children's Hospital. All family members were clinically evaluated by reviewing patient history, performing physical examinations, and consulting medical records. The parents and elder brother were healthy. The affected twins and their unaffected parents and elder brother were enrolled in the study. The probands of this family were the 9-month-old twins (II-2 and II-3). They were hospitalized due to hypoxia after birth and diagnosed with TOF using a Philips Epiq5 ultrasonic diagnostic instrument (S8-3 probe, frequency 3.0–8.0 MHz) according to American Society of Echocardiography criteria. The father took an echocardiogram as well. Peripheral blood samples were collected from the patients and their family members. This study was approved by the Ethics Committee of Wuhan Children's Hospital, and informed consent was obtained from the parents of this family.

### Whole Exome Sequencing

Genomic DNA was extracted from the peripheral blood of the family members using a QIAamp DNA Blood Mini Kit (Qiagen, GER) according to the manufacturer's instructions. The quality of the DNA samples was then measured by NanoDrop2000 (Thermo Scientific, United States).

Exome sequencing was performed on the five members of this family. In brief, the subjects' exomes were captured using an Agilent SureSelect Human All Exon V6 Enrichment kit (Agilent, Santa Clara, CA, United States) and then sequenced on a NovaSeq platform (Illumina, San Diego, CA, United States) according to the manufacturer's guides, generating 150-bp paired-end reads. All reads were mapped to the NCBI human reference genome (hg19) using Burrows-Wheeler Alignment version 0.7.9a (http://bio-bwa.sourceforge.net). Single nucleotide variants (SNVs) and indels were detected using Genome Analysis Toolkit version 3.5 (https://gatk.broadinstitute.org/hc/en-us) and annotated using ANNOVAR (https://annovar.openbioinformatics.org/en/latest/user-guide/download/) based on splice site, intronic, exonic, 5' UTR, 3' UTR, intergenic, upstream, or downstream locations. For prioritization, missense, non–sense, frameshift, non-frameshift, or splicing site variants with a minimum allele frequency (MAF) of more than 0.1% in the East Asians and the total population in the gnomAD (https://gnomad.broadinstitute.org) database were removed.

### *In silico* Analysis

The pathogenicity of the variants was predicted using online software programs: Sorting Intolerant From Tolerant (SIFT; http://sift-dna.org), PolyPhen-2 (http://genetics.bwh.harvard.edu/pph2/), and MutationTaster (http://www.mutationtaster.org). The missense variants predicted to be deleterious by at least two of the above tools were selected for future confirmation and analyses. *In silico* prediction studies were then carried out for the selected variant using other different online available tools to further illustrate the effect on the protein function. We also used CLC Sequence Viewer 8 software to analyze the conservation of the mutated site in various species.

### Sanger Sequencing Validation

A suspected disease-causing variant of the *MYOM2* gene, c.3097C > T, was validated in the affected twins by standard Sanger sequencing. Primer5 software was used to design the polymerase chain reaction (PCR) primers specific to the regions of the variants in *MYOM2*. The primer sequences are shown in Table 1. The healthy parents and the elder brother were then sequenced for the segregation analysis of the variant.

**Table 1:** *MOYM2*-specific primers used for Sanger sequencing.

**ID**	**Sequence**	**Length (bp)**
*MYOM2*- F	CTGTTGCTAATCCGAGTG	1,095
*MYOM2*- R	GATGGGATCTCCTTATGTT	

### Protein Modeling

The amino acid sequence of human *MYOM2* was obtained from the NCBI (NP_003961.3). The wild-type and variant structures of the *MYOM2* were built using the SWISS-MODEL. PyMOL software was used to represent structural figures.

## Results

### Clinical Descriptions

**Figure 1:**
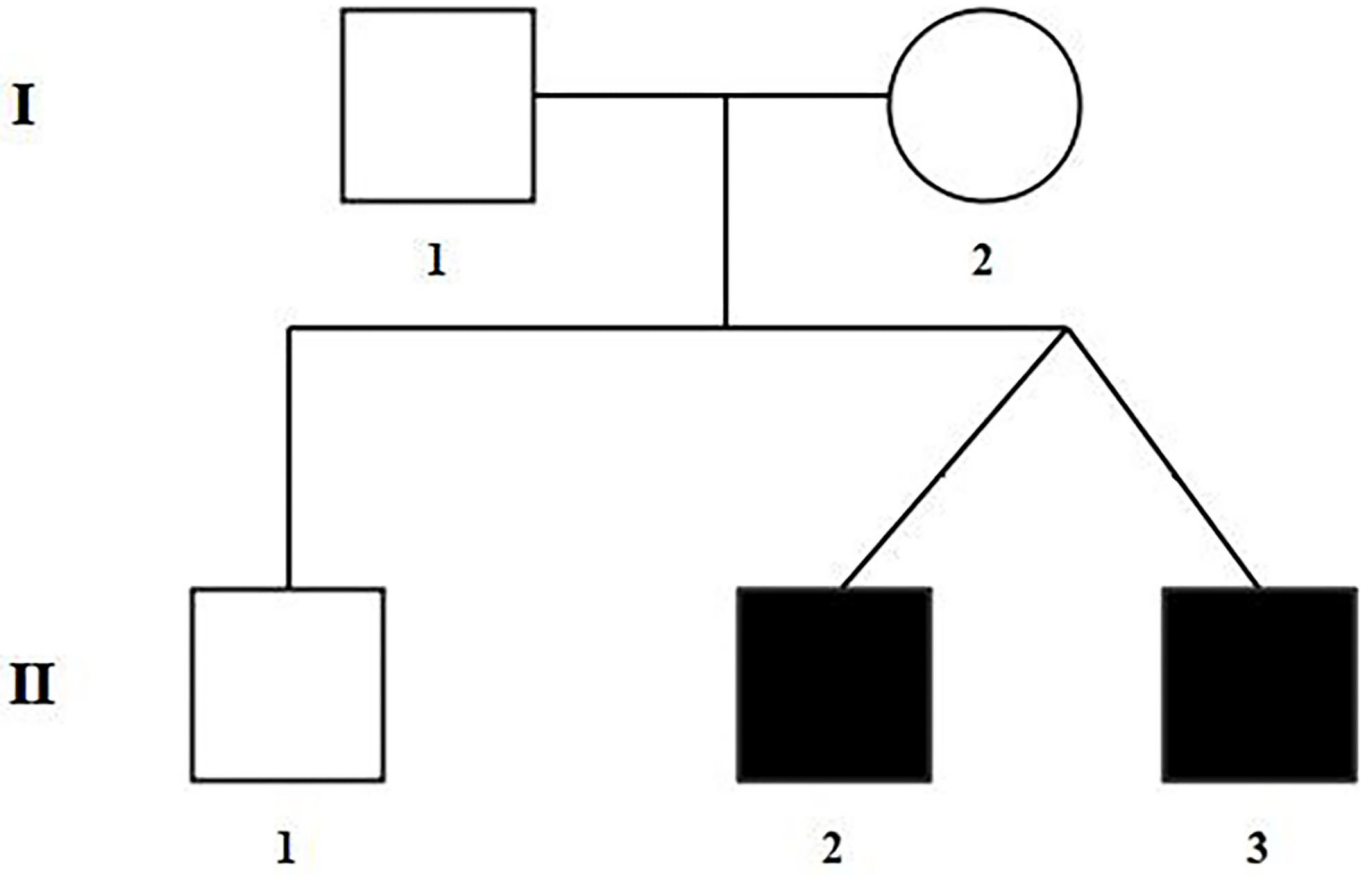
Pedigree of the family with TOF. The filled black symbols represent the affected members.

**Figure 2:**
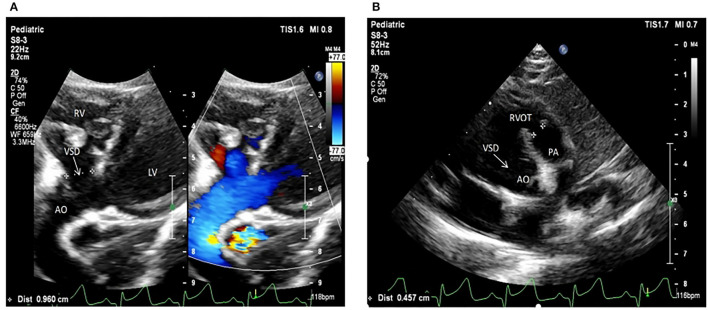
Echocardiogram images from patients II:2 and II:3 showing the apex of the heart. **(A)** The ultrasonogram for the elder twin (II:2). **(B)** The ultrasonogram for the younger twin (II:3). LV, left ventricle; RV, right ventricle; Ao, aorta; RVOT, right ventricular out ow tract; PA, pulmonary artery.

The selected Chinese family included healthy non-consanguineous parents (I-1and I-2), a healthy elder brother (II-1), and two affected male twins (II-2 and II-3) (Figure 1). The probands (II-2 and II-3) were diagnosed by echocardiography at 9 months and 6 days old as having TOF. No other developmental abnormalities were found during a series of examinations, including a physical examination. They had smooth breathing, no obvious cyanosis of the face and lips, and were not in any distress. As shown on the ultrasonogram for the affected elder twin (Figure 2A), the position of the heart, aortic ventricular connections, and aorta were normal. The aortic arch had three branches with normal initial position and arrangement. The aortic wall was smooth, the lumen was unobstructed, and the inner diameter was about 15.6 mm. However, the outflow tract of the right ventricle was narrow and about 4.0 mm wide. And interruptions occurred in interventricular septa, a few left-to-right shunt signals were detected in the lower atrial septum and a small amount of regurgitation was detected on the tricuspid valve. For his twin brother as shown on the ultrasonogram (Figure 2B), the position of the heart, aortic ventricular connections, and aorta were normal, and the valve morphology, structure, and opening and closing were normal. However, interruptions occurred in interventricular septa. The pulmonary artery was stenosed and the outflow tract of the right ventricle was narrow and about 4.8 mm wide. There was a localized convex posterior to the upper part of the pulmonary artery. Based on these results, the twins were diagnosed with TOF. In addition, the echo results of the father illustrated that the shape, structure, wall thickness, and function of the heart were all normal (Supplemental Figure 1). Furthermore, none of the other family members exhibited TOF.

### Exome Sequencing and Co-Segregation Analysis

**Figure 3:**
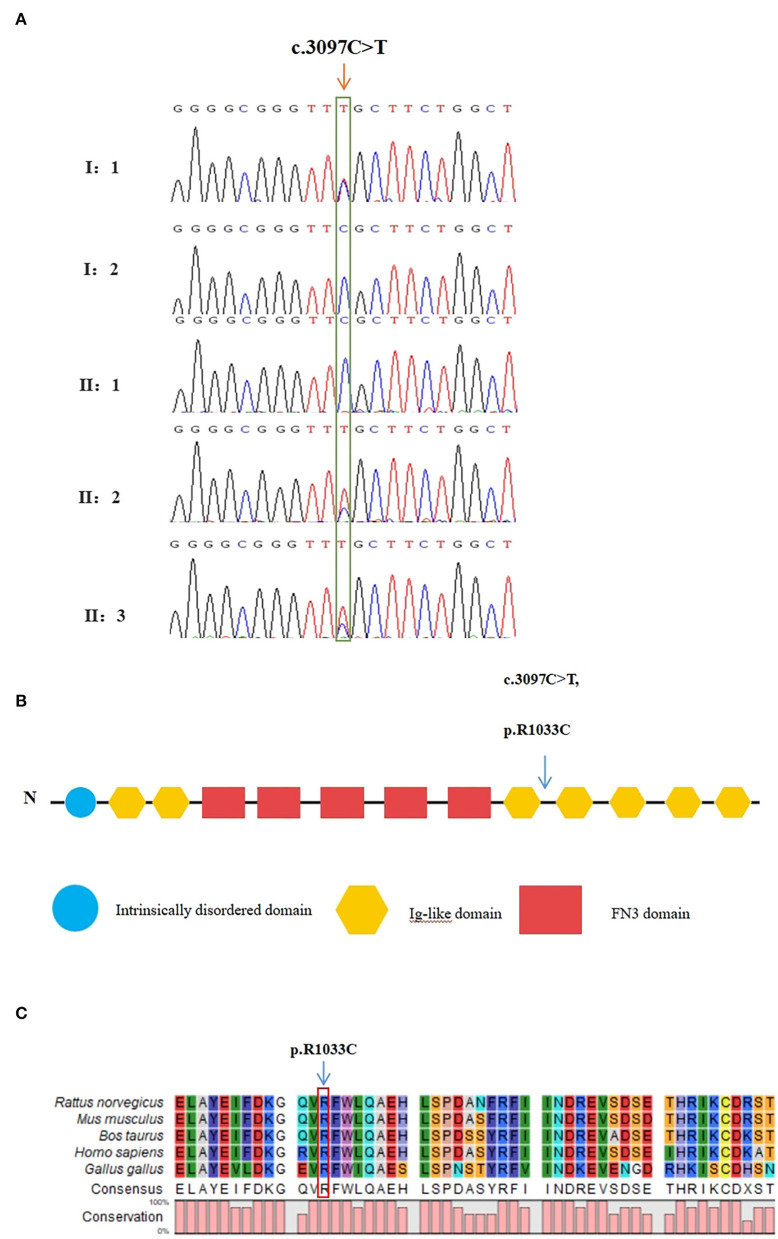
Validation of the missense variant of *MYOM2* in the family with TOF. **(A)** Sanger sequencing results from the probands, their brothers, and their unaffected parents. The heterozygous variant in the *MYOM2* gene was identified in the twins, but not in their healthy brother. **(B)** The location of the variant in the protein structure of *MYOM2*. The arrow denotes the mutated site. **(C)** Amino acid alignment of the *MYOM2* protein from several organisms. The position of Arg1033 residue (highlighted by a red box) was highly conserved among different species.

The raw data of each subject was about 12G. The mean depth of the target region was 100X and the mean coverage of the target region was 99.8%. Following rigorous filtering for rare, predicted pathogenic variants shared in the twins, and the sequencing data from the elder brother (II:1) which was used to exclude any variants that were present in the unaffected brother, we found that the affected twins did not share any homozygous or compound heterozygous mutations of previously reported genes, as well as biallelic variants of novel genes. In addition, they also did not share any de novo variants. We subsequently screened 48 heterozygous, likely deleterious variants that were shared in the twins, including 15 variants inherited from the father and 33 variants inherited from the mother (see Supplemental Table 1). By examining the literature for known associations related to heart diseases, for example, whether the gene variants have been reported in patients with congenital heart diseases, whether mutant mice showed abnormal cardiac development, or whether they were involved in biological processes associated with cardiac development, we finally identified a heterozygous missense variant in exon 25 of *MYOM2* gene, c.3097C>T (p.R1033C) as a possible genetic cause of TOF. The Sanger sequencing confirmed that this variant was present in the affected twins. The healthy father had a heterozygous status of this variant, however, the unaffected mother and brother did not carry this variant (Figure 3A).

### *In silico* Analysis of the *MOYM2* Missense Variant

The minor allele frequency of the observed variant was assessed in East Asian and global populations using the GnomAD database and ChinaMap database (http://www.mbiobank.com/) which included deep whole genome sequencing data from 10,588 Chinese participants (22). The variant is extremely rare in both the East Asian and global populations (0.00016 and 0.000024, respectively). Furthermore, it is absent in the ChinaMap database. The missense variant of *MYOM2* was predicted to be highly damaging to the function of the *MYOM2* protein using multiple online prediction tools including SIFT, PolyPhen2, PROVEAN, MutationTaster, and CADD (Table 2). The variant site is near the Ig-like domain (Figure 3B). In addition, the alignment of *MYOM2* amino acid sequences in different species showed high conservation of arginine at position 1033 (Figure 3C). These results suggest that this variant is highly pathogenic, and we hypothesized that this rare heterozygous missense variant of *MYOM2* may be the cause of TOF in this affected family.

**Table 2:** Pathogenicity prediction of the identified mutation (c.3097C>T,p.R1033C) using multiple online *in silico* tools.

**No.**	***In silico*** **analysis tools**	**Prediction**	**Score**
1	SIFT	Damaging	0.0
2	Polyphen-2	Probably_damaging	1.0
3	MutationTaster	Disease_causing	1.0
4	FATHMM-MKL	Damaging	0.986
5	CADD	Damaging	35
6	DANN	Damaging	0.999
7	PROVEAN	Damaging	−7.24
8	VEST3	Damaging	0.963
9	ClinPred	pathogenic	0.983
10	GenoCanyon	Damaging	1.00
11	INPS-MD	Stabilitychange	−0.53

### Effect of Mutation on Protein

**Figure 4:**
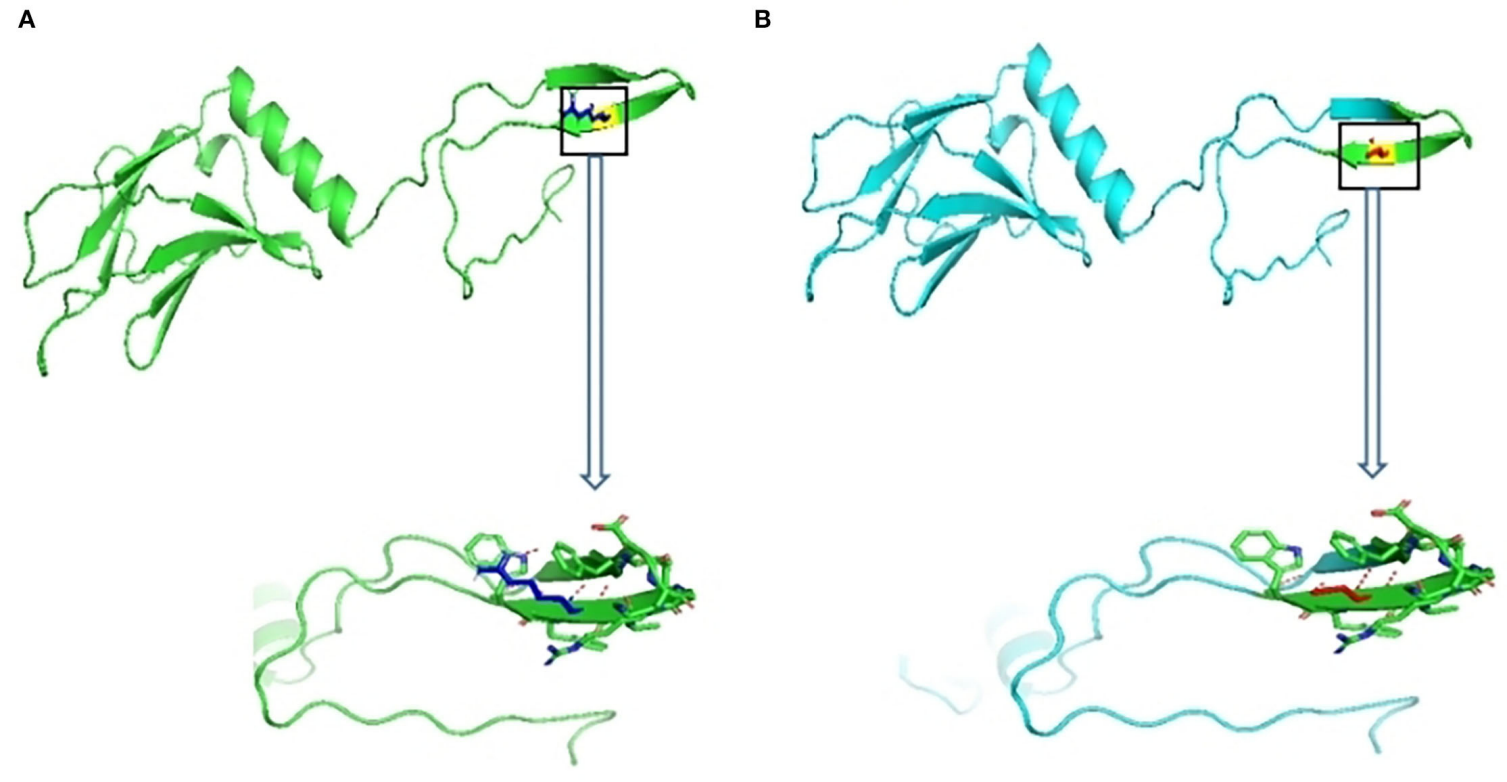
Wild-type and variant structure of *MYOM2* protein. **(A)** Wild-type *MYOM2* predicted 3D structure. **(B)** Mutation *MYOM2* predicted 3D structure. Supplemental Figure 1 Echo images from the twins' father. The above 4 figures were taken from Cardiac ultrasound results. These figures illustrate the shape, structure, wall thickness, and function of the heart were all normal.

The 3D structure of *MYOM2* protein was already created using the SWISS-MODEL program. To investigate the missense variant effect on protein, schematic structures of the WT (left) and the mutant (right) amino acids are shown in Figure 4. Compared with the 3D structure of WT protein, the mutant protein showed a different steric hindrance of the residue (the new residue has a smaller size), which may lead to protein misfolding, resulting in pathogenicity.

## Discussion

In this study, we enrolled a Chinese TOF family with two affected twins and identified a rare deleterious variant of the *MYOM2* gene, c.3097C>T (p.R1033C) in the family by whole exome and Sanger sequencing. We speculated that this variant may be the genetic cause of TOF.

As a highly heterogeneous disease, previous studies have suggested that rare potential pathogenic variants of genes related to the heart development, such as Filamin A (*FLNA*), Kinase Insert Domain Receptor (*KDR*), and *NKX2.5* are associated with the occurrence of TOF (12, 18, 19, 23). Recently, several variants in the sarcomere genes such as Myosin Heavy Chain 7 (*MYH7*) and Myosin Binding Protein C3 (*MYBPC3*) have been identified in patients with heart diseases (24–27). However, to date, the correlations between genetic variants of sarcomere genes and TOF have not been fully established.

The Myomesin gene family comprises three members including *MYOM1, MYOM2*, and *MYOM3*, and encodes the proteins of sarcomere which play important roles in the development of heart and skeletal muscles (28, 29). They all include fibronectin type III (FN3) and immunoglobulin type II (Ig) like domains, acting as crosslinkers for the neighboring thick filaments of myosin in the M-band. The Ig-like domains can be found in several diverse protein families and are involved in a variety of functions, including cell-cell recognition, cell-surface receptors, muscle structure, and the immune system (30). These three myosin subtypes are also related to the contractile properties of different fiber types. While *MYOM1* is expressed in all striated muscles, *MYOM3* is expressed in skeletal muscle intermediate fiber types, and *MYOM2* is expressed in the adult heart and fast fibers (31–33). Previous studies have shown that variations in *MYOM1* and *MYOM3* could cause cardiac abnormalities, such as hypertrophic cardiomyopathy and dilated cardiomyopathy (34, 35). Moreover, Pehlivan et al. (2019) found that the loss of function of *MYOM2* can result in the termination of gestation of the affected fetus, with cardiac and arthrogryposis findings, but this does not occur with loss of function of *MYOM1* or *MYOM3* (36). Auxerre-Plantie et al. (2020) identified three heterozygous deleterious variants of *MYOM2* (c.590C > T, p.A197V; c.3320G > C, p.G1107A; and c.3904A > G, p.T1302A) which is located on the Ig-like domains and one deleterious variant of *MYOM2* (c.2119G > A, p.A707T) which was located on the FN3 domain in TOF patients (21). Furthermore, Tang et al. (2022) also identified two compound heterozygous variants of *MYOM2* in Chinese TOF patients and found the top enriched cluster of heart development contained GO terms that were related to the development of cardiac muscle and morphogenesis, including genes encoding the myomesins (*MYOM1* and *MYOM2*) by functional enrichment analysis (20). These results might provide several genetic clues to the association between *MYOM2* and heart development.

*MYOM2* is located on chromosome 8p23.3, contains 37 exons, and encodes a 1,465–amino acid protein which is expressed in the human heart muscle and is a major structural component of the M-band (33, 37, 38). The sarcomere with two transverse structures, the Z-disk, and the M-band, is the Myofibrils' basic unit. Myofibrils have been reported to mediate skeletal and cardiac muscle contraction in vertebrates and invertebrates. Therefore, alterations of sarcomere proteins may influence the contractile performance of the heart and skeletal muscle (35). In addition, cardiomyocytes derived from induced pluripotent stem cells of healthy individuals and TOF patients reveal that *MYOM2* is expressed during cardiac differentiation (39). Furthermore, *MYOM2* is also expressed in embryonic and adult mouse hearts (40). Although there are no studies that mention the mammalian animal model for exploring the exact role of *MYOM2* in the heart development, given the advantages of the fly model for studying the genetics of human disease mechanisms (41), Auxerre-Plantie et al. (2020) aimed to clarify the potential cardiac function of *MYOM2* using *Drosophila* (21). They found that its partial loss of function or moderate cardiac knockdown resulted in cardiac dilation, whereas severely reduced function caused an increase in sarcomere myosin protein, which is clearly involved in the development of the heart. In addition, as it was shown that *MYOM2* physically interacts with *MYH7*, which is known to be associated with the development of CHDs in *vitro*, the CG14964, the fly ortholog for *MYOM2*, and Mhc, the fly ortholog for *MYH7*, could also interact at the genetic level *in vivo* (25, 26, 42). Furthermore, they also identified some heterozygous missense variants of *MYOM2* in TOF patients. The above findings indicate that *MYOM2* might be a candidate gene for TOF.

In this study, we performed WES on all five members of a TOF-affected family to identify novel potential pathogenic variants and genes based on rigorous bioinformatic analyses. By applying several filtering processes, we identified a heterozygous missense variant, c.3097C > T (p.R1033C), in *MYOM2* in the affected twins. Furthermore, Sanger sequencing confirmed that, while the variant was present in the affected twins and the proband's father, who did not have any abnormal cardiac phenotypes, the proband's healthy brother did not carry the variant. These results showed that this variant was transmitted from an unaffected father indicating incomplete penetrance. A previous study has reported rare heterozygous variants of *MYOM2* in TOF patients without co-segregation analysis (21). Some rare heterozygous missense variants in *NOTCH1*, a known disease-causing gene, have also been reported in the TOF patients and some of these variants are inherited from their unaffected parents (7). The incomplete penetrance is in keeping with the complex genetic etiology of non-syndromic TOF, in which families segregating the condition in a Mendelian fashion are rarely encountered and genetic background, except for the environmental factors, can be inferred to play significant roles. In addition, the missense variant identified in *MYOM2* is extremely rare in East Asian populations and the overall human population, based on the genome database archived in gnomAD and is absent in the ChinaMap database (www.mBiobank.com). Furthermore, the mutated site is highly conserved among mammals, and the variant was predicted to be deleterious by multiple online software programs. Interestingly, this mutated site is near to the previously reported rare deleterious variant of *MYOM2*. Therefore, the heterozygous missense variant that we found may have caused the TOF by affecting *MYOM2* protein function in this family.

In summary, we identified a novel heterozygous missense variant of *MYOM2* (NM_003970.4:c.3097C>T:p.R1033C) as a possible genetic contributor to familial TOF in a Chinese population. This finding increases our knowledge of the etiology of TOF by identifying a possible causative gene variant, which should be considered when diagnosing this disease. However, the exact role of this variant for TOF has not been clearly explained. Therefore, further studies are required to elucidate the exact mechanism of the association between the missense *MYOM2* variant and TOF by performing functional experiments *in vitro* and *in vivo*, and studies with larger sample sizes are needed to identify more variants of *MYOM2* in different populations.

## Data Availability Statement

The original contributions presented in the study are included in the article/Supplementary Material, further inquiries can be directed to the corresponding authors.

## Ethics Statement

The studies involving human participants were reviewed and approved by the Ethics Committee of Wuhan Children's Hospital. Written informed consent to participate in this study was provided by the participants' legal guardian/next of kin. Written informed consent was obtained from the minor(s)' legal guardian/next of kin for the publication of any potentially identifiable images or data included in this article.

## Author Contributions

All authors contributed to the study conception and design. The research was designed by JW, LG, and MP. Ultrasound images were provided by HX, LW, and XF. Data analysis and interpretation were performed by JW, CW, and BW. The validation experiment was performed by TL. The first draft of the manuscript was written by CW and JW. All authors commented on early versions of the manuscript, contributed to the article, and read and approved the submitted version.

## Funding

This work was supported by the project of the Elevate Project of Medical Basis, School of Basic Medical Sciences, Capital Medical University, 2021; Basic Science Promotion Project of Capital Medical University; the Central Government to Guide Local Scientific and Technological Development (2018ZYYD063); and the Science and Technology Project of Wuhan Health Commission (EX20E06).

## Conflict of Interest

The authors declare that the research was conducted in the absence of any commercial or financial relationships that could be construed as a potential conflict of interest.

## Publisher's Note

All claims expressed in this article are solely those of the authors and do not necessarily represent those of their affiliated organizations, or those of the publisher, the editors and the reviewers. Any product that may be evaluated in this article, or claim that may be made by its manufacturer, is not guaranteed or endorsed by the publisher.
