# Supplementary material for: Case Report: Tetralogy of Fallot in a Chinese Family Caused by a Novel Missense Variant of MYOM2
Source: Front Cardiovasc Med. 2022 Jul 7;9:863650. doi: 10.3389/fcvm.2022.863650 (PMC9300848; doi:10.3389/fcvm.2022.863650)
Supplement: Supplementary file 1 [file Table_1.DOCX]

**Table S1. Heterozygous rare variants shared by TOF patients.**

| Gene | Chr | Variant | Frequency | Prediction | | |  | Inheritance |
| --- | --- | --- | --- | --- | --- | --- | --- | --- |
|  |  |  |  | SIFT | PolyPhen2 | MutationTaster | CADD |  |
| ADPGK | 15 | NM_031284:c.T1256C:p.I419T | 0.000024/0.00028 | D | D | D | 24.5 | Paternal |
| CPAMD8 | 19 | NM_015692:c.T3065G:p.V1022G | absent | D | P | D | 23.2 | Paternal |
| ESRP1 | 8 | NM_001034915:c.C1253T:p.S418L | 0.000044/0.00028 | D | D | D | 35 | Paternal |
| FAM193B | 5 | NM_001190946:c.G2264A:p.R755H | absent | D | D | D | 32 | Paternal |
| GEM | 8 | GEM:NM_005261:c.7_8del:p.L3fs | absent | NA | NA | NA | NA | Paternal |
| HDGFRP2 | 19 | NM_001001520:c.2015_2171del:p.*672fs | absent | NA | NA | NA | NA | Paternal |
| INPP5F | 10 | NM_001243194:c.C571T:p.R191X | absent | NA | NA | D | 40 | Paternal |
| IQGAP3 | 1 | NM_178229:c.A49G:p.T17A | 0.000036/0.00049 | D | P | D | 26.5 | Paternal |
| KRT10 | 17 | NM_000421:c.1683_1684insAGCTCCGGCGGCGGATACGGCGGCGGCAGC:p.S562delinsSSGGGYGGGSS | absent | NA | NA | NA | NA | Paternal |
| MYOM2 | 8 | NM_003970:c.C3097T:p.R1033C | 0.000024/0.00016 | D | D | D | 35 | Paternal |
| PKN1 | 19 | NM_002741:c.G2071A:p.V691M | absent | D | D | D | 33 | Paternal |
| POU4F2 | 4 | NM_004575:c.C991G:p.R331G | absent | T | D | D | 22.1 | Paternal |
| TRIM3 | 11 | NM_001248006:c.C365T:p.T122M | 0.000068/0.00033 | D | D | D | 23.2 | Paternal |
| VPS13C | 15 | NM_017684:c.G6882C:p.E2294D | absent | D | D | D | 28 | Paternal |
| TTN | 2 | NM_003319:c.C30581T:p.A10194V | absent | T | D | D | 23.6 | Paternal |
| ADGRF2 | 6 | NM_153839:c.T458A:p.I153K | absent | D | D | D | 27.8 | Maternal |
| AMOTL1 | 11 | NM_001301007:c.G1897A:p.D633N | absent | D | D | D | 32 | Maternal |
| ATP1A2 | 1 | NM_000702:c.A1589G:p.E530G | 0.000016/0.00011 | D | B | D | 23.2 | Maternal |
| CAND2 | 3 | NM_012298:c.G2761A:p.A921T | absent | D | D | D | 25.2 | Maternal |
| CHST15 | 10 | NM_001270764:c.G671A:p.R224H | 0.0000041/0.000056 | D | B | D | 23.7 | Maternal |
| CRB3 | 19 | NM_139161:c.C320T:p.P107L | absent | T | D | D | 18.13 | Maternal |
| CTDSP2 | 12 | NM_005730:c.T317C:p.I106T | 0.000008/0 | D | P | D | 25.3 | Maternal |
| DDC | 7 | NM_001242888:c.G956A:p.R319H | absent | D | D | D | 23 | Maternal |
| PRSS3 | 9 | NM_001197098:c.G395C:p.C132S | absent | D | P | D | 22.6 | Maternal |
| FRG2C | 3 | NM_001124759:c.463delG:p.G155fs | absent | NA | NA | NA | NA | Maternal |
| HHIP | 4 | NM_022475:c.C716T:p.S239L | 0.000085/0.00099 | D | D | D | 34 | Maternal |
| HLA-DRB1 | 6 | NM_002124:c.C239G:p.T80R | 0.00013/0 | D | D | D | 23.4 | Maternal |
| IGSF3 | 1 | NM_001542:c.G760A:p.D254N | absent | D | D | D | 32 | Maternal |
| INPP5B | 1 | NM_001297434:c.C704G:p.S235C | absent | T | D | D | 26.3 | Maternal |
| KMT5A | 12 | NM_020382:c.T995C:p.L332P | absent | D | D | D | 26.9 | Maternal |
| MMP17 | 12 | NM_016155:c.G640A:p.G214S | 0.000042/0.00022 | D | P | D | 13.21 | Maternal |
| MST1L | 1 | NM_001271733:c.811dupG:p.A271fs | absent | NA | NA | NA | NA | Maternal |
| MUC4 | 3 | NM_018406:c.4909_4910insGCCCTTCCTCAGCATCCACAGGTCACGCCACCCCTCTTCCTGTCACCA:p.N1637delinsSPSSASTGHATPLPVTN | absent | NA | NA | NA | NA | Maternal |
| NELL1 | 11 | NM_001288714:c.G2072T:p.R691L | absent | D | D | D | 24 | Maternal |
| OR52B6 | 11 | NM_001005162:c.A584G:p.H195R | absent | D | D | P | 16.18 | Maternal |
| PCDHA12 | 5 | NM_018903:c.T2309C:p.M770T | absent | D | D | D | 21 | Maternal |
| PCSK1 | 5 | NM_000439:c.C844T:p.R282W | 0.000024/0 | D | D | D | 29.9 | Maternal |
| PDE6C | 10 | NM_006204:c.C487T:p.H163Y | 0.00006/0.00033 | D | P | D | 24.8 | Maternal |
| PDHX | 11 | NM_001135024:c.C520A:p.R174S | absent | D | D | D | 33 | Maternal |
| PGR | 11 | NM_000926:c.G1475T:p.R492L | absent | D | D | D | 32 | Maternal |
| PSPH | 7 | NM_004577:c.G268A:p.G90S | absent | D | D | D | 27.1 | Maternal |
| SDF4 | 1 | NM_016176:c.C668T:p.S223L | 0.000049/0.0001 | D | D | D | 26.8 | Maternal |
| SLC24A1 | 15 | NM_001301031:c.C1502T:p.A501V | absent | D | D | D | 29.7 | Maternal |
| SNCAIP | 5 | NM_001308105:c.A446G:p.D149G | absent | D | D | D | 27.2 | Maternal |
| STON1 | 2 | NM_001198594:c.T1850A:p.V617D | 0.00006/0.00082 | D | D | D | 28.6 | Maternal |
| UGT2B7 | 4 | NM_001074:c.G1192A:p.D398N | 0.000028/0 | D | P | D | 23.2 | Maternal |
| ZNF598 | 16 | NM_178167:c.C2237T:p.A746V | absent | T | D | D | 24 | Maternal |
| ZNF77 | 19 | NM_021217:c.120_130del:p.L40fs | absent | NA | NA | NA | NA | Maternal |

^$^ Frequency in overall population/East Asian population in gnomAD.

Abbreviation: NA, not available; T, Tolerable; B, benign; P, Possibly_damaging; D, damaging.
