# Supplementary figures and images for: Case Report: Tetralogy of Fallot in a Chinese Family Caused by a Novel Missense Variant of MYOM2
Source: Front Cardiovasc Med. 2022 Jul 7;9:863650. doi: 10.3389/fcvm.2022.863650 (PMC9300848; doi:10.3389/fcvm.2022.863650)

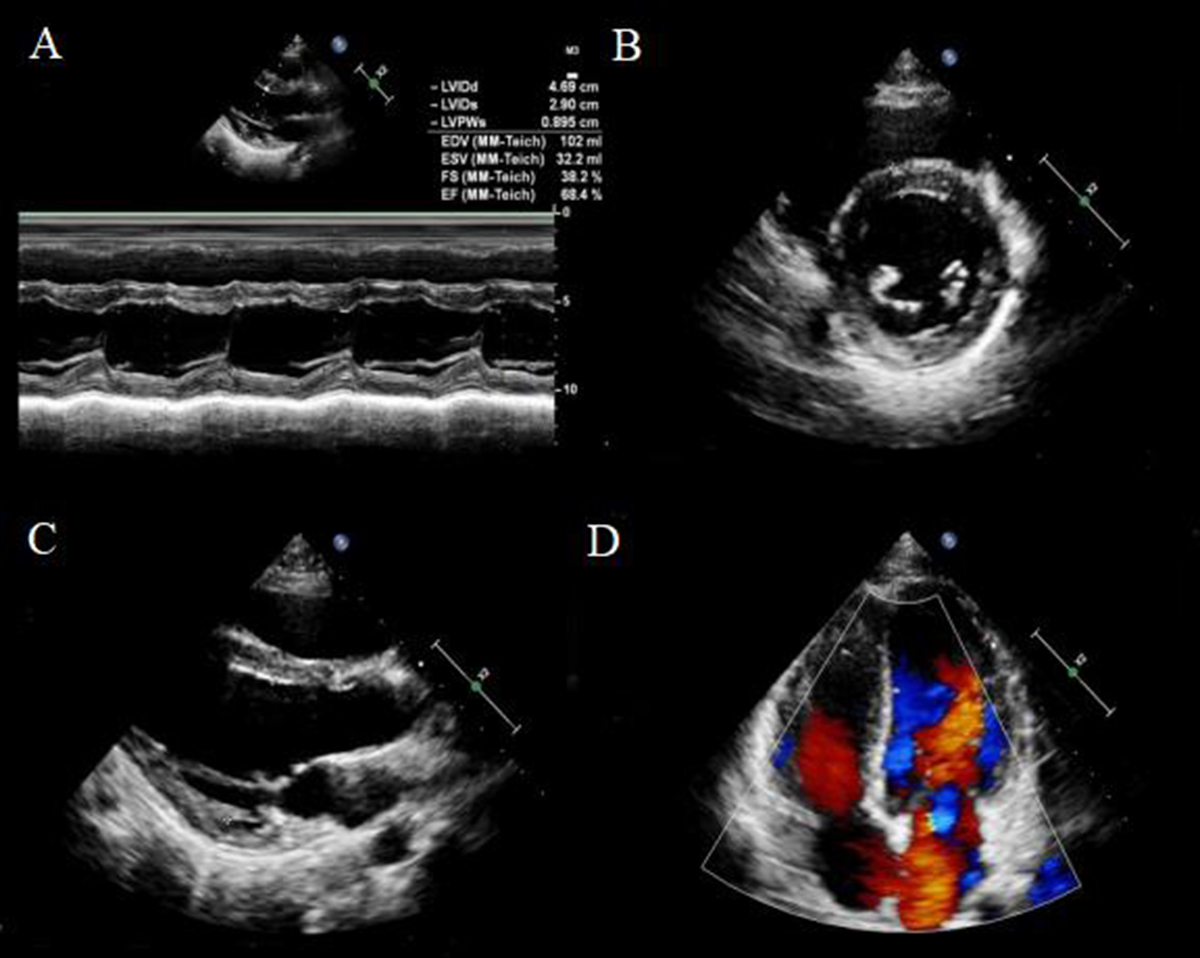

Supplement: Supplementary file 2 [file Image_1.JPEG]
